# Experience of learning from everyday work in daily safety huddles—a multi-method study

**DOI:** 10.1186/s12913-022-08462-9

**Published:** 2022-08-30

**Authors:** Karina Wahl, Margaretha Stenmarker, Axel Ros

**Affiliations:** 1 Department of Paediatrics, Region Jönköping County, 55185 Jönköping, SE Sweden; 2 Department of Clinical and Experimental Medicine, Linköping University, Linköping, Sweden; 3 Futurum/Department of Paediatrics, Region Jönköping County, Jönköping, Sweden; 4 Department of Paediatrics, Institute of Clinical Sciences, The Sahlgrenska Academy at the University of Gothenburg, Gothenburg, Sweden; 5 Jönköping Academy for Improvement of Health and Welfare, Jönköping University and Futurum, Jönköping Region Jönköping County, Jönköping, Sweden

**Keywords:** Safety huddles, Safety-II, Improvement work, Resilience, Safety culture measurement

## Abstract

**Background:**

To reduce patient harm, healthcare has focused on improvement based on learning from errors and adverse events (Safety-I). Daily huddles with staff are used to support incident reporting and learning in healthcare. It is proposed that learning for improvement should also be based on situations where work goes well (Safety-II); daily safety huddles should also reflect this approach. A Safety-II-inspired model for safety huddles was developed and implemented at the Neonatal Care Unit at a regional hospital in Sweden. This study followed the implementation with the research questions:

Do patient safety huddles with a focus on Safety-II affect the results of measurements of the patient safety culture? What are the experiences of these huddles amongst staff? What experiences of everyday work arise in the patient safety huddles?

**Methods:**

A multi-method approach was used. The quantitative part consisted of a questionnaire (151 items), submitted on four different occasions, and analysed using Mann Whitney U-test and Kruskal Wallis ANOVA-test. The qualitative data were analysed using thematic content analyses of interviews with staff (*n* = 14), as well as answers to open questions in the questionnaires.

**Results:**

There were 151 individual responses to the questionnaires. The response rates were 44% to 59%. For most comparisons, there were no differences. There were minor changes in patient safety culture measurements. A lower rating was found in December 2020, compared to October 2019 (*p* < 0.05), regarding whether the employees pointed out when something was about to go wrong. The interviews revealed that, even though most respondents were generally positive towards the huddles (supporting factors), there were problems (hindering factors) in introducing Safety-II concepts in daily safety huddles. There was a challenge to understanding and describing things that go well.

**Conclusions:**

For patient safety huddles aimed at exploring everyday work to be experienced as a base for learning, including both negative and positive events (Safety-II); there is a need for an open and permissive climate, that all professions participate and stable conditions in management. Support from managers and knowledge of the underpinning Safety-II theories of those who lead the huddles, may also be of importance.

**Supplementary Information:**

The online version contains supplementary material available at 10.1186/s12913-022-08462-9.

## Background

To improve patient safety and reduce patient harm, healthcare providers have focused on routines for risk management and systems to identify and remedy deficiencies. Despite all efforts made in patient safety work, the number of healthcare injuries is still high [1]. In the work on systems for incident reporting, too much focus has been on collecting reports, with less on learning, improvement, and the social processes around incidents [2]. More focus on these latter factors may help in reducing the rate of incidents, as the actions and efforts tried previously have not brought about the desired improvements in healthcare.

It has been suggested that patient safety work is facing a paradigm shift [3]. Instead of only looking at incidents and accidents (termed a Safety-I perspective), it is also important to focus on learning from what employees do well, and how they adapt to the varying and difficult conditions in which they work (Safety-II) [4]. In a Safety-II perspective, safety work should be based on understanding how individuals act and perform their work in everyday life, so that things most often go right, and then based on that understanding, try to support work so that safety is improved [4].

A system is said to be resilient when it can achieve what it is intended to accomplish, both under expected and unexpected conditions [5]. Resilience engineering (RE) is a field that studies system resilience, and entails the conscious design of a system to make it resilient [6]. In resilience, four potentials are suggested as important in a system, namely: to be able to respond—to know what to do in different situations; to monitor—to know what to look for and measure it;—to learn from all experiences; and to anticipate—to know what to expect in the future [7]. The Safety-II perspective is necessary in order to describe how these potentials are expressed in a system (for example a workplace in healthcare), and to develop an understanding of how work in ordinary life is accomplished, taking into account all variability that there is [4].

The literature on resilience and Safety-II and their application in healthcare is expanding, but mainly based on case studies and from theoretical and methodological perspectives. It has been argued that there is a need for more empirical research in the field [8]; and to design interventions and operationalise changes based on RE principles and to measure their effectiveness [9]. A review of RE literature highlighted the need for studies on hindering factors for implementation of Safety-II and RE principles in practice, as well as the need to provide practical guidance to managers on how to design and operate resilient organisations [10]. 

It has been argued that a Safety-II and resilience approach to learning from everyday work stimulates staff participation in the learning process, which supports patient safety improvement [11]. There are existing models for reflection in daily huddles [12–14]. The Green Cross model is commonly used in healthcare in Sweden with the purpose of learning from adverse events [14]. In the Green Cross model, staff meet in a daily huddle to discuss and reflect on patient safety incidents and risks for patient harm that have occurred during the day in order to support incident reporting and learning – a Safety-I approach. The Resilient Performance Enhancement Toolkit (RPET), is suggested as a tool that supports daily conversations at a workplace to promote learning and improvement based on everything that happens in ordinary work – a Safety-II approach. Within an improvement work, a daily safety huddle based on a Safety-II approach, called the Green Line, was developed and introduced at the neonatal intensive care unit (NICU) at the Ryhov county hospital in Jönköping, Sweden in October 2018. The Green Line was developed in close cooperation with Erik Hollnagel while he was writing up his RPET whitepaper [15]. The overall aim of the improvement work in the NICU is to improve patient safety through introducing a Safety-II approach to learning from everyday work.

The Green Line is a tool to support daily conversations and to promote learning and improvement based on everything that happens in ordinary work in a patient safety huddle. There are emerging descriptions on the use of different methods to use a Safety-II approach to support learning in healthcare settings based on written accounts of experiences of everyday work [11, 16]. Patient safety huddles offer a simpler and more direct way of communication than written accounts, and might thus be a more convenient way to promote learning and improvement from ordinary work.

The aim of this study was to describe experiences of changing the focus of patient safety work from only learning from deviations (Safety-I) to also learning from when things go well (Safety-II) during patient safety huddles at a hospital unit, using the research questions:

- Do reflections with a focus on Safety-II in patient safety huddles affect the results of measurements of the patient safety culture conducted using questionnaires?
- What experiences of the Green Line with a Safety-II approach have staff had?
- What experiences of everyday work arise in the patient safety huddles that can be classified according to the potentials defined in resilience engineering; respond, monitor, learn, anticipate?

## Methods

**Fig. 1:**
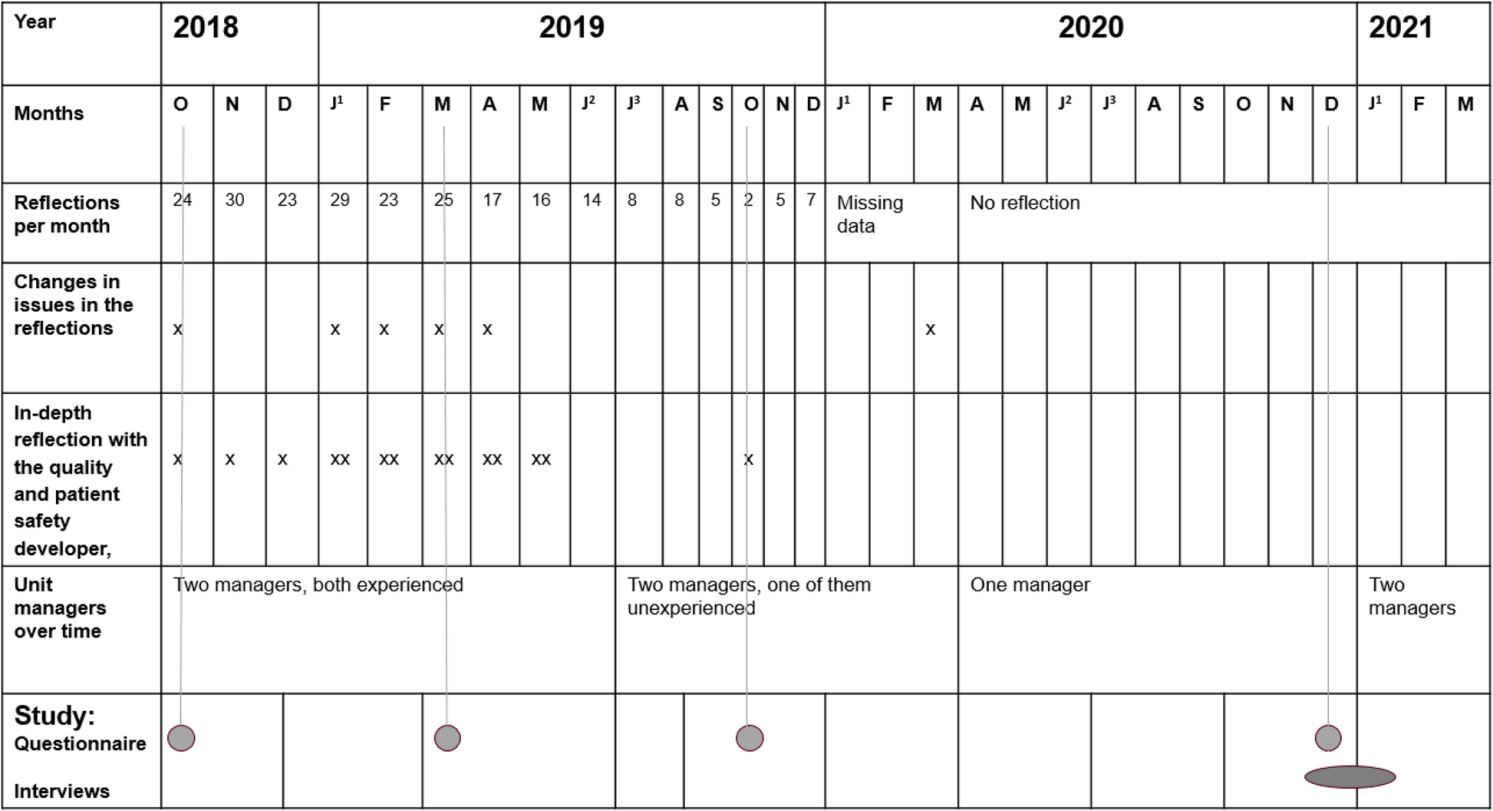
Study and improvement work. Description of changes in the local context and the design of the study and the improvement work. Description of when the survey and interviews in the study were conducted linked to the situation at the unit. J1 = January, F = February, M = March, A = April, M = May, J2 = June, J3 = July, A = August, S = September, O = October, N = November, D = December

This study follows the implementation of the Green Line method at an NICU with a multi-method approach, using separate quantitative and qualitative analyses. The study was originally planned as an improvement work and the instruments used for the study were chosen based on the methods used in the improvement work in combination with data from it. A description of how the study relates to changes in the improvement work is described in Fig. 1.

### Context/settings

The NICU studied is part of the department of paediatrics at a regional hospital in the southern part of Sweden. There are 16 patient beds, and approximately 40 nurses, 20 assistant nurses and seven doctors are employed. The unit cares for newborn infants and premature infants born from gestational week 27, in need of intensive care. Two nurse managers and a consultant physician are responsible for management of the unit. In each work shift, the management has appointed a bed and staff coordinator. A quality and patient safety developer at the department of paediatrics supports improvement works at the NICU.

### The Green Line improvement work at the NICU

The NICU management team decided to introduce the Green Line method to improve and support patient safety work at the unit. An inter-professional improvement group with nurses and assistant nurses introduced and led the implementation, starting in October 2018. All employees were introduced to the Green Line model and trained in how to use it, including the theoretical concepts of resilience and Safety-I and -II. To facilitate the implementation of the Green Line, the inter-professional improvement group had support from the quality and patient safety developer and the unit management team. Regular meetings with the improvement group were planned to follow up on and change the improvement work when needed. Improvements were tested and changed according to the PDSA model for improvement work: Plan, Do, Study, and Act [17] (Additional file 1). Professor Erik Hollnagel was present at a huddle at the beginning and participated in a meeting with the improvement group to provide input on the improvement work. In discussions within the improvement group together with the quality and patient safety developer the general questions to support Safety-II-inspired safety huddles suggested in RPET [15] were adapted to suite the workplace and the intentions of the improvement work. The questions were open with the intention to use follow-up questions: “How did you manage that?”, “Can you describe more?” Based on the experiences of the patient safety huddles, questions were changed over time so that the reflections in the huddle were perceived as valuable for learning. Some examples are: “How have we succeeded today?”, “What have we done to ensure that all children receive food?” To sharpen the patient safety huddles focus topics for reflection were introduced, for example encounters with parents and staff, and breaks to provide relief from work.

The Green Line reflections was planned to be 5–10 min long in the afternoon and all staff that could attend were invited to take part. During the meeting, short notes were taken by one of the participants in the patient safety huddle, to insure that ideas for improvement could be collected. The bed and staff coordinator led and facilitated the reflection. Sometimes this task was performed by members of the improvement group, when they were on duty. At the end of the reflection, the meeting was classified according to its main content: discussion and learning based on a Safety-II perspective, discussion and learning based on a negative event (Safety-I), only reporting of a negative event, or no reflection at all. A classification, based on a colour scheme, was used as is described in Erik Hollnagels RPET whitepaper [15]. To support the learning process and ensure deeper reflection, the quality and patient safety developer participated once or twice a month in the safety huddles. This person also wrote a summary every month of the number of participants, profession, colour classification and what potentials the conversations could be classified within. The summary was shown to the employees to further strengthen their awareness of the role of resilience and the Safety-II approach. During the improvement work, the questionnaire described in the quantitative method section was used to follow up on the patient safety culture at the unit. One statement was added so that the questionnaire had items to cover all four potentials described in resilience. The question added was formulated “At my workplace, we have routines and working methods that allow us to be prepared for challenges that may arise”, and is not reported in this study.

### Quantitative method

#### Data collection and analysis

The questionnaires used in the improvement work to investigate the patient safety culture formed the basis for the quantitative analyses of this study. The questionnaire contained 11 items phrased as statements and was distributed and repeated four times during the study period. This questionnaire has been developed by the Swedish association of local authorities and regions, together with researchers and experts in patient safety, as a simplified tool for patient safety culture assessment [18, 19]. The answer alternatives in the questionnaire use a five-point Likert scale, 1–5 (1 = I totally disagree, 5 = I fully agree) (Additional file 2). One open-ended question to capture the views of the individual was added in the last three surveys, namely “Comments on the Green Line reflections?” The questionnaires were sent out to all employees by work e-mail on four occasions, October 2018, March 2019, October 2019, and December 2020, along with a request for voluntary participation (Additional file 3). The answers were collected using the web survey system esMaker [20], which enables anonymous participation.

#### Statistical analysis

Results are presented as numbers, frequencies, medians and 25th and 75th percentiles when suitable. A Mann Whitney U-test was used to analyse differences between two groups. A Kruskal Wallis ANOVA-test was used when there were comparisons between more than two groups. The adjusted *P*-value is shown. The following were compared: occasion for the surveys, profession and years of employment at the unit (≤ 10 year, > 10 year), The *P*-value is reported as * < 0.05, ** < 0.01, *** < 0.001. The data analysis was generated using SPSS version 25 (IBM Corp., Armonk, NY, USA).

### Qualitative method

Two sources were used for the qualitative analysis: the answers to the open question in the questionnaire “Comments on the Green Line reflections? Enter positive as well as negative views”, and semi-structured individual interviews with employees (*N* = 14). These interviews were performed from December 2020 to January 2021.

**Fig. 2:**
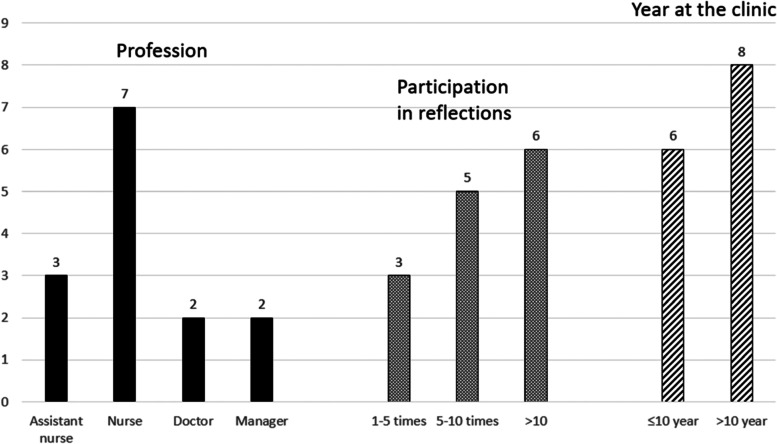
Interviewees – demographic information. Description of demographic information regarding the interview participants, their profession, number of times they participated in reflections and number of years at the unit

In the interviews, employees were asked about participation by a strategic selection with maximum variability sampling to achieve a wide distribution by profession and by years of employment, and by convenience (Fig. 2). Information was provided in writing (Additional file 4) and orally. The interviews were conducted by KW, an experienced quality and patient safety developer, under the guidance of AR and MS who are experienced researchers. The interviews were recorded, a question guide was used (Additional file 5) and notes were taken. At the end of the interview a summary was made by KW and additional comments and changes could be made. The interviews lasted 5—24 min (mean 14 min) and were conducted during working hours. The interviews were transcribed verbatim prior to the analysis and were anonymised. The results were analysed on a group level, and are presented with de-identified illustrative quotes.

Three different qualitative analyses were performed.

- I). A deductive analysis of the interviews to provide an answer to the question of what experiences of everyday work are highlighted in the reflections. The four potentials described in resilience—respond, monitor, learn and anticipate—were used as a framework in the analysis [7].
- II and III). Two different inductive thematic content analyses were performed separately on the interviews and the open answers in the questionnaire to the question about experiences of the Green Line approach. The content analysis according to Malterud is based on themes, codes and meaning units and is an analysis of different types of data, such as interviews and written text [21].

The first analysis was made by KW, and an initial version of themes and codes was developed. The analysis was then adjusted and verified in discussions with AR and discussed and confirmed with MS. The results were validated through feedback from the interviewees.

## Results

The results section contains a description of changes during the improvement work as well as quantitative and qualitative results.

### Changes and results in the improvement work

During the study period the improvement work changed over time. The changes were made by the improvement group, based on experiences of the implementation and according to the PDSA model. These changes and some contextual factors are displayed in Fig. 1. The frequency of safety huddles changed during the studied period; the initial intention was to have them daily, but that was intentionally changed to twice a week. But even so, not all planned safety huddles were performed as planned. Changes were made to the questions used to support the reflections and also to how often the patient and safety developer participated in the safety huddles. There were also changes in management; in one period there was one manager instead of two. On average, eight to ten professionals participated in each patient safety huddle. During the Covid-19 pandemic no intentional changes were made in the safety huddles, except that they were moved from a small room to a larger one. Examples of the monthly summaries intended to further strengthen awareness of resilience and safety-II is displayed in Additional file 6, with the number of participants, profession, colour classification and what potentials of resilience the conversations could be classified within.

Examples of practical improvements at NICU as a result of the safety huddles are provided in Additional file 7.

### Quantitative result / results of survey

There were 151 individual responses to the questionnaires, submitted on four different occasions. The response rates were 44% to 59% (Tables 1, 2, 3). The results are presented in Tables 4, 5, 6, 7. For most comparisons, there were no differences. For some there were significant differences, but with small differences in specific numbers. The statement “I point out when I think something is about to go wrong” was valued significantly lower in December 2020 compared to October 2019 (Table 4). The following statements: “In my workplace, we always act on the risks we see”, “I dare to talk about my mistakes” and “I would feel safe if a close relative was cared for at my workplace” were rated significantly lower by those who had worked > 10 years compared to those who had worked ≤ 10 years (Table 5). Physicians indicated to a higher degree their ability to adapt and collaborate than other professions did (Table 6). There was no difference in comparisons of the statement “The Green Line reflections lead to learning” within the entire staff group i.e. not between professions, or according to years employed in the last three surveys (Table 7).

**Table 1:** Survey responses, by four occasions for questionnaires

Questionnaires	Responses/quantity	Response rate
October 2018	41/69	59%
March 2019	37/69	54%
October 2019	39/69	56%
December 2020	34/77	44%

Survey responses on four occasions. Questionnaires, number of responses, number of questionnaires and response rate in %

**Table 2:** Survey responses by profession

Profession	Number	%
Doctor	6	4%
Nurse	101	67%
Assistent nurse	41	27%
Other occupational group	3	2%

Survey responses by professions number and %

**Table 3:** Survey responses by years of employment at the unit

Year at the unit	Number	**%**
≤ 10 Year	71	47%
> 10 Year	79	52%

Survey responses by year at the unit, number and %. (One person had not specified year at the unit, 150 answers to this question)

**Table 4:** Survey responses to questionnaires on four occasions

**Comparison/occasion**		**2018 October (N41)**	**2019 March (N37)**	**2019 October (N39)**	**2020 December (N34)**
3. My boss provides conditions for conducting safe care	Median 25th/75th	5 4/5	4 4/5	4 4/5	5 4/5
4. In my workplace, we learn from what works well	Median 25th/75th	4 4/5	4 4/5	4 4/4	4 4/5
5. In my workplace, we always act on the risks we see	Median 25th/75th	4 4/4.75	4 4/5	4 4/4	4 4/4.25
6. In my workplace, improvements are always made after negative events	Median 25th/75th	4 4/4	4 4/5	4 4/4	4 4/4
7. I point out when i think something is about to go wrong	Median 25th/75th	4 4/5	5 4/5	5 4/5	4* 4/5
8. I dare to talk about my mistakes	Median 25th/75th	4 4/5	4 4/5	4 4/5	4 4/5
9. I am always well received at my workplace when i need help	Median 25th/75th	4 4/5	5 4/5	4 4/5	4 4/5
10. At my workplace, we have a well-functioning collaboration with other units	Median 25th/75th	4 3/4	4 4/4	4 3/4	4 3/4
11. At my workplace, we adapt the work so that safety is maintained when conditions change	Median 25th/75th	4 4/4	4 4/4	4 4/4	4 4/4
13. I would feel safe if a close relative was cared for at my workplace	Median 25th/75th	5 4/5	4 4/5	5 4/5	4 4/5
14. At my workplace, we offer parents / relatives the opportunity to be involved in our patient safety work	Median 25th/75th	3 3/4	4 3/4	4 3/4	4 3/4

The answer alternatives in the questionnaire were a five-point Likert scale, 1–5 (1 = I totally disagree, 5 = I fully agree) (Additional file 1). N is number of answers. Median, percentile 25th, percentile 75th, * = *P* < 0.05, ** = *P* < 0.01, *** = *P* < 0.001

* is December 2020 in comparison with October 2019

**Table 5:** Survey responses to questionnaires by years of employment at the unit

**Comparison / year at the unit**		** ≤ 10 Year** **(N71)**	** > 10 Year** **(N79)**
3. My boss provides conditions for conducting safe care	Median 25th/75th	4 4/5	4 4/5
4. In my workplace, we learn from what works well	Median 25th/75th	4 4/5	4 4/5
5. In my workplace, we always act on the risks we see	Median 25th/75th	4 4/5	4* 4/4
6. In my workplace, improvements are always made after negative events	Median 25th/75th	4 4/4	4 4/4
7. I point out when i think something is about to go wrong	Median 25th/75th	5 4/5	4 4/5
8. I dare to talk about my mistakes	Median 25th/75th	5 4/5	4* 4/5
9. I am always well received at my workplace when i need help	Median 25th/75th	4 4/5	4 4/5
10. At my workplace, we have a well-functioning collaboration with other units	Median 25th/75th	4 3/4	4 3/4
11. At my workplace, we adapt the work so that safety is maintained when conditions change	Median 25th/75th	4 4/5	4 4/4
13. I would feel safe if a close relative was cared for at my workplace	Median 25th/75th	5 4/5	4*** 4/5
14. At my workplace, we offer parents / relatives the opportunity to be involved in our patient safety work	Median 25th/75th	4 3/4	4 3/4

The answer alternatives in the questionnaire were a five-point Likert scale, 1–5 (1 = I totally disagree, 5 = I fully agree) (Additional file 1). N is number of answers. Median, percentile 25th, percentile 75th, * = *P* < 0.05, ** = *P* < 0.01, *** = *P* < 0.001

**Table 6:** Survey responses to questionnaires by profession

**Comparison/profession**		**Other occupational group (N3)**	**Assistant nurse (N41)**	**Doctor** **(N6)**	**Nurse** **(N101)**
3. My boss provides conditions for conducting safe care	Median 25th/75th	5 5/5	4 4/5	5 4.75/5	4 4/5
4. In my workplace, we learn from what works well	Median 25th/75th	5 -	4 4/4.5	4,5 4/5	4 4/5
5. In my workplace, we always act on the risks we see	Median 25th/75th	4 4/4	4 4/5	4 4/5	4 4/5
6. In my workplace, improvements are always made after negative events	Median 25th/75th	4 4/4	4 3/4	4 4/5	4 4/4
7. I point out when i think something is about to go wrong	Median 25th/75th	5 5/5	4 4/5	4 3.75/5	5 4/5
8. I dare to talk about my mistakes	Median 25th/75th	4 -	4 4/5	4,5 4/5	4 4/5
9. I am always well received at my workplace when i need help	Median 25th/75th	5 5/5	4 4/5	5 3.75/5	4 4/5
10. At my workplace, we have a well-functioning collaboration with other units	Median 25th/75th	4 4/4	4 3/4	4*^1^ 4/4.25	4 3/4
11. At my workplace, we adapt the work so that safety is maintained when conditions change	Median 25th/75th	4 4/4	4 4/4	5**^1^**^2^ 4.75/5	4 4/4
13. I would feel safe if a close relative was cared for at my workplace	Median 25th/75th	5 -	4 4/5	5 4.75/5	4 4/5
14. At my workplace, we offer parents / relatives the opportunity to be involved in our patient safety work	Median 25th/75th	3 3/3	4 3/4	3.5 2.75/5	4 3/4

The answer alternatives in the questionnaire were a five-point Likert scale, 1–5 (1 = I totally disagree, 5 = I fully agree) (Additional file 1). N is number of answers. Median, percentile 25th, percentile 75th, * = *P* < 0.05, ** = *P* < 0.01, *** = *P* < 0.001. *^1^ and **^1^ is doctor in comparison with assistant nurses. **^2^ is doctor in comparison with nurse

Due to the small number of respondents in the group, other occupational group, 25th and 75th is not reported in question 4, 8, and 13

**Table 7:** Survey responses to the question, “The green line reflections lead to a learning”

**Comparison/occasion**	**2019 March (N37)**	**2019 October (N39)**	**2020 December (N34)**	
Median 25TH/75TH	4 3/4	4 3/4	4 3/4	
Comparison/profession	**Other occupational group** **(N3)**	**Assistant nurse** **(N41)**	**Doctor** **(N6)**	**Nurse** **(N101)**
Median 25TH/75TH	4 4/4	3,5 3/4	4 4/4	4 3/4
Comparison/year at the unit	** ≤ 10 year** **(N71)**	** > 10 year** **(N79)**		
Median 25TH/75TH	4 3/4	4 3/4		

The answer alternatives in the questionnaire were a five-point Likert scale, 1–5 (1 = I totally disagree, 5 = I fully agree) (Additional file 1). The comparison is made by three occasions, by profession and by year at the unit respectively

N is number of answers. Median, percentile 25th, percentile 75th, * = *P* < 0.05, ** = *P* < 0.01, *** = *P* < 0.001

### Qualitative results

Demographic data for each one of the interviews, are presented in Table 8.

**Table 8:** Demographic information of interviewed persons, their profession, years of employment at the unit and number of occasions they participated in the safety huddles

Participants	Profession	Year at the unit	Participation in safety huddles
P1 ^a^	Assistant nurse	> 10 Year	5–10 times
P2 ^a^	Nurse	> 10 Year	> 10 times
P3 ^a^	Nurse	> 10 Year	> 10 times
P4	Doctor	> 10 Year	5–10 times
P5 ^a^	Nurse	≤ 10 year	> 10 times
P6 ^a^	Nurse	≤ 10 year	5–10 times
P7 ^a^	Manager	≤ 10 year	> 10 times
P8	Nurse	≤ 10 year	1–5 times
P9 ^a^	Doctor	> 10 Year	1–5 times
P10	Manager	> 10 Year	5–10 times
P11^a^	Nurse	> 10 Year	> 10 times
P12^a^	Assistant nurse	> 10 Year	5–10 times
P13	Assistant nurse	> 10 Year	> 10 times
P14	Nurse	≤ 10 year	1–5 times

^a^ In the article cited interviewed persons

### Experiences of everyday work

The experiences reported were categorised according to the potentials described in resilience, i.e. respond, monitor, learn, and anticipate. There were many examples from learn and respond, fewer from anticipate and only one example from monitor.

### Respond

The respondents gave examples of how they adapted to the conditions of work, for example by distributing patients and staff more evenly over the ward. NICU staff, both new and experienced ones, said that training was necessary to be able to respond properly to unusual situations.

‘When a very sick child arrives, suddenly large parts of the staff disappear to care for that child. How did we then manage to take care of all the other patients? A parent could take care of their child in a way that we had not planned. Someone from the general paediatric unit came to help us…” (P3).

“Everyone knows this except me…..then you realise that almost everyone wanted training…. some things are unusual…..”(P6).

This staff member indicated that she thought she was the only one interested in these issues, but the safety huddles indicated that her concerns were shared by her colleagues. The respondents believe that it is important to highlight examples in the safety huddles on how things work out, and how they have managed and responded to different situations.

### Monitor

Only one experience emerged that can be traced to the monitor potential. The respondent describes the importance of staff being with the patients and observing changes in the patient´s status so they do not miss anything. “You should not miss anyone…you have to observe, it is how I think…we observe them” (P12). This is an experience that is at the individual patient level; there were no experiences reported of the potential monitor at the system level.

### Learn

Experiences were described of learning from activities in daily work, from mistakes and learning from colleagues but it was described as difficult to learn from situations that had been resolved.

“Narcanti, we have two different dilutions. First a mistake was discussed in the Green Line and then it was close to a mistake again ….. it was a nurse who reacted before the drug was given, we had talked about that kind of problem before (in the safety huddles)” (P7).

“….we highlight the good examples and learn from them …….what have we done well today?—Well, we have substituted for each other in coffee breaks, that was good …..There were no real learning opportunities” (P5).

The learning in the safety huddles were mainly from negative events, very few from things that had gone well, when problems were resolved. It seems difficult to get an in-depth reflection on why situations were resolved in a good way.

### Anticipate

The respondents experienced that everyday work was becoming more and more unpredictable and complex, and some examples came up in the safety huddles of how they anticipated problems to be better prepared to deal with them.

“….plan your day with the person you work with…lunch and everything…who should go first…otherwise…no one has a break” (P1).

“Say you need help, instead of saying today I did not get a break…don’t think that you should sort it out yourself” (P1).

Work is often unpredictable, but it is important to plan the day when it is busy, for example, who should go on a break first and when, and ask for help instead of complaining afterwards. So even if it was not intentional in the format of the safety huddles, anticipation and preparedness for difficulties in the coming work shifts were subjects raised according to the respondents.

### Reflections on the “Green Line method”

In both the analysis of interviews and the open answers from the questionnaire, two themes emerged, "Supporting factors" and "Hindering factors”. There was also an overlap between the codes; hence the results are reported together since both sources were reflections on the same phenomenon. Three and two codes respectively were found for each theme (Table 9, Additional file 8).

**Table 9:** Themes and codes from the analysis of the interviews and from the questionnaire

Theme	Code
Supporting factors	Seeing benefits with reflection
	Learning from what happens
	Finding improvements for a rewarding reflection
Hindering factors	Seeing difficulties with reflection
	The impact of the work climate

### Supporting factors

The theme “Supporting factors” describes the codes "Seeing benefits with reflection", "Learning from what happens" and "Finding improvements for a rewarding reflection".

### Seeing benefits with reflection

All respondents mentioned that it was valuable to have reflections in general. The safety huddles offer an opportunity for those who do not speak out in any other context in daily work or staff meetings at the unit. There is an opportunity to get confirmation that they have done the right thing and they can get input from others´ solutions.

On the basis of good examples from the reflections in the huddles it can be easier to address negative things. The safety huddles also support creating common values and cohesion in a unit where the employees are working in different areas.

“I really think it's great …. It’s not only mistakes that should be noticed, you can learn a lot from each other, everyone has different experiences….” (P7). "It's such a scattered department you may not even see others throughout the shift… I think there will be a little more cohesion in the group because of reflections…" (P11).

It is said that it is valuable to reflect on what happened during a day, and the safety huddles improve the cohesion in the working group. Without reflections in the huddles it is difficult to get the opportunity to share experiences because the unit is divided into different care rooms.

### Learning from what happens

It was experienced as difficult to talk about and learn from things that went well; these positive experiences are taken for granted, and it was easier to talk about something negative. But the view was expressed that it was good to highlight and concretise things that went well so others could learn from them. There were also notes that negative comments were not taken seriously. Sometimes it was perceived as taboo to talk about when something went wrong.

“It is difficult because…for everything in life really, if you do not hear anything, then it is probably often good… you are only told the bad things” (P1).

“No one dared to say anything that was negative” (free comment survey 2020).

This may depend on expectations that only positive events should be addressed in the huddle, or on the working climate and the role of openness. Hence, it is difficult to focus on what goes well when nothing negative happens and the challenge of talking about both the positive and negative things instead of just the negative things.

### Finding improvements for a rewarding reflection

The respondents pointed out suggestions for improvements to make reflections in the patient safety huddles more useful and valuable. The role of the safety huddle leader was important; he/she needed to be interested, direct the conversations and believe that the reflection was important. It was good that the quality and patient safety developer sometimes facilitated the huddles. It was important to develop the method without changing to new methods or giving new names to the method; the method was just the tool. The huddles needed to be varied and inspiring, not static with too limited conversation. It was good to vary the questions for example with different focus topics.

“… Someone who is clear about the purpose and who agrees with the purpose, I think so, not just someone who is set to lead that reflection” (P9).“…they became more inspired… the (reflection) leader must have the ability to angle the questions” (P2). “It became more lively when we started using focus topics” (P11).

The safety huddles should be regular, short, objective and with the right focus. There were different opinions about the frequency: every day, twice a week or on demand. The safety huddles needed to be planned in the schedule, so that doctors could also participate, since they were better if all professions participated. It was also regarded as important that the managers were involved and supportive.

“…that the managers try to participate and are interested and also think it is important” (P3).

It seems necessary to clarify the purpose of the safety huddles and to find ways to spread lessons from them. The leadership role of the safety huddle is important, as well as the ability of the leader of the huddle to get in-depth reflections. It is important to involve all, to schedule all professions so that they can participate in safety huddles.

### Hindering factors

The theme “Hindering factors” describes the codes: "Seeing difficulties with reflection" and "The impact of the work climate”.

### Seeing difficulties with reflection

Some respondents said that safety huddles got stuck in the format, so that the format was more important than what was reflected on, which was perceived as inhibiting, and there was a need to clarify the purpose of the huddles. There were no learning opportunities and it was hard to keep them serious and focused. There was more focus on staff working hours and breaks than on the actual task of creating good care. When the reflection was based on what went well, it was often the same things that came up, which was not useful. It was difficult to find times that suited all employees to attend the huddles. Things that needed to come up were not discussed, and things that came up were not carried forward, since those who could answer were not present, which was frustrating.

“..There was often a lot of repetition, it was the same thing. And everything that becomes the same thing becomes very boring” (P2).

“Feedback is given but stays there …it may need to reach other people … or make improvements… often it stays in the small group… and the challenge may continue to bother you” (P6).

Difficulties with the safety huddles were described; it was hard to keep the reflections serious and focused, there were no learning opportunities and the purpose was not clear. It was difficult to make improvements when not all professions participated.

### The impact of the work climate

The respondents felt that how easy it was to dare to reflect openly varied, depending on the situation and the constellation of participants. Comments emerged that sometimes the atmosphere during the safety huddles was not inviting and the conversations were superficial. There was a desire for an open and permissive climate but the experience was that this was not always the case. The work in the unit meant that the employees were scattered inside different care rooms and did not see each other during the working day if there is no opportunity to gather, for example during a safety huddle. There was a great work experience and skill in the group; many had worked for a long time and it was difficult as a new person to dare to talk, and it was difficult to join the working group. The managers were important for creating the climate and supporting the Green Line reflections.

“We should have a slightly more open climate in our department….the attitude of some in the staff group may be….judges a little too easily sometimes” (P6). “…when you are new, you are invisible” (P5). “Managers must be involved in the Green Line project and support an open climate…..” (P3).

The impact of the work climate and the difficulties that exist when employees were scattered in individual care rooms throughout the day were described. It was difficult to join the working group when there was great work experience and skill in the group. A safety huddle could lead to improved cohesion and community in the group but needed support from the managers.

## Discussion

This study evaluates the introduction of Safety-II inspired reflections in patient safety huddles for staff at a hospital ward. Thus, it is an attempt to draw empirical knowledge from interventions designed to operationalise changes based on Safety-II and resilience engineering principles [8, 9]. Most respondents were positive towards safety huddles generally, but it was found that to really lead to learning and improvement, the format and support for a Safety-II inspired reflection needs to be developed and the purpose needs to be clarified further. There were different opinions about what was easy or difficult when performing the safety huddles. Our findings suggest these matters depended on the situation, who took part in the safety huddle and who led it. There were minor changes in some aspects of patient safety culture measurements over time during the study period. In the experiences discussed in the safety huddles, there were examples of the system potentials of resilience: learn, respond and anticipate, but only one of the potential monitor.

It was perceived difficult to reflect on and learn from what was going well, the Safety-II perspective. The literature on learning in a Safety-II perspective is still sparse. In one study on an intervention based on written reports on things that had gone well, the number of reports was smaller than expected, problems getting staff engaged on a wide scale were discussed, and it was concluded that learning from how things go well is a simple yet compelling concept [16]. Our study supports this reflection; Safety-I learning took precedence in relation to Safety-II in the safety huddles at the NICU, even though both can co-exist. We normally “see” when an adverse event takes place, but we do not “see” when an adverse event does not take place, when things go well [15]. Healthcare professionals are trained to see and report adverse events [2]. If they do not see what is going well, it is difficult for them to understand and describe it. In a study of nurses' experiences of the incident reporting culture after implementation of the Green Cross method, it was found that it was not good to focus only on things that went wrong, and it was suggested that health care would benefit from learning both from successes and errors [22]. In the present study it was found necessary that staff understood that shifting focus from Safety-I to II should include learning from both Safety-I and II perspectives [23].

One goal of the Green Line reflections was to support learning. Adults learn what they experience as meaningful, they take as much responsibility as they are interested in, and they do not get involved if they do not see any meaning to what they are learning [24]. Leadership is important to create good conditions and a permissive climate for learning [25]. Our study supports the view that the role of managers is important; reflections in patient safety huddles need support from clear leadership by the managers at the unit, and the purpose of the safety huddles needs to be constantly clarified [14]. Managers at the clinical level are central to the system’s capacity for expressing resilience but they need more models and training in how to approach their work [25]. Managers need to continuously follow up an intervention to reinforce commitment for a change to be fully accepted and established in the workplace [26]. A development-oriented leadership where managers support employees' learning as part of development can be successful. The manager's role is to clarify expectations, prioritise development issues, create resources, and to follow up [27]. In this study, there were shortcomings in how the improvement work was followed up in the long term.

The impact of the work environment is central. Psychological safety, a belief that one will not be punished or humiliated for speaking up with ideas, questions, concerns or mistakes, is important in a workplace to support tolerance and openness [28]. Tolerance and openness in a workplace helps patient safety huddles to be perceived as rewarding so that they support learning based on reflections on both negative and positive events. The desire is to improve the work environment based on experiences expressed in the reflections; but there also needs to be a good work environment to encourage people to risk sharing experiences. There was a significant change from October 2019 and December 2020 regarding whether the employees pointed out when something was about to go wrong (with a lower rating in December 2020). Apart from aspects of psychological safety that were expressed in the interviews, this may also be explained by the management changes, and changes in format of and support for the safety huddles. In an NICU, collective learning based on safety reporting and accumulated knowledge in prioritizing of and performing the work may be difficult, since the work is performed inside individual care rooms, as has been pointed out by Hybinett et al. [25]. To see each other and share experiences may contribute to psychological safety. Safety huddles for all staff can offer an opportunity to share experiences and increase knowledge in a unit where work is dispersed, as in a NICU.

In this study, the Safety-II inspired safety huddles were found sometimes to be worthwhile and sometimes not. They turned out differently depending on who led the reflections, their experience in doing so, their knowledge of the theoretical background, and their ability to get an in-depth reflection. How open and tolerant the participants and the work climate were, was also of importance. Furthermore, it was appreciated if someone else from outside the unit, for example the quality and patient safety developer, sometimes led the reflection to ensure it was deeper. In a study of the Green Cross method in healthcare (i. e. Safety-I), Schwarz et al. also found that the leadership role in the meeting is important [14]. It helps to have supporting questions and open questions, and the questions need to be varied. In addition, it is good if all professions participate; in our study it was reported that physicians did not attend the safety huddles as regularly as other professions. In another study on the effect of the Green Cross method on incident reporting the participation of physicians was also highlighted [29]. For learning according to Safety-II to happen, the importance of reflecting on everyday practice, and ensuring that such reflection is routinely carried out in practice, is important [30]. Our findings support this; schedule planning is needed and the safety huddles have to occur regularly. The safety huddles need to be tailored to the staff's needs and have an actual impact on improving their work to be experienced as rewarding and valuable. There is a need to develop methods to spread lessons and support improvements based on positive events in the same manner as from negative events, i. e. to explore everyday work [31].

The respondents experienced that everyday life was becoming increasingly complex and there was a need to adapt to different situations. In complex enterprises such as modern health care it is necessary to make pragmatic adaptations to changing contexts, including in the introduction of improvement interventions [32]. To support the development and testing of improvements in complex healthcare systems, PDSA cycles are well established. In these, ideas are transformed into action, the actions are tested and studied to learn and to improve them, and this is continued in a cycle, for continuous improvement [17, 33]. PDSA is an established improvement tool in the NICU, and it was used initially to support the Green Line work, but not thoroughly over time. It may have been valuable to continue the PDSA cycles until the Green Line reflections had been satisfactory established.

All four potentials that have been suggested to describe a resilient system [7], were exemplified in the reflections, but to varying degrees. Communication, for example the safety huddles in this study, can contribute to the four potentials, but do not directly contribute to resilient performance [34]. There were more examples reported in the interviews from the potentials learn and respond, while there were fewer from anticipate and only one from monitor. There is possibly a greater propensity for healthcare professionals to act, than to be actively aware of what they can expect from the future and from measurements. This can be exemplified by the work situation at an NICU, as has been described by Hybinette et al. The focus in an NICU is on unpredictable factors such as acute admissions, where one has to quickly readjust plans and actions and where the inflow of emergency patients may have the highest priority [25]. One possible way forward to better highlight and describe examples of expression of resilient capacity might be to use a number of pre-defined issues for the reflections which can reflect and draw attention to all four potentials.

### Method discussion and limitation

Different methods were used with the aim of capturing aspects of the experience and impact of the Green Line method. This project was originally designed as an improvement project, not a research study. Had it been so, another approach to evaluating possible effects on patient safety culture would have been chosen. Measuring the patient safety culture using questionnaires can be useful. Hospitals that have good results in patient safety measurements also have lower numbers of adverse safety events; but further research is needed to investigate the relationship between the measured safety culture and the improvement in clinical safety [35]. The instrument chosen for evaluation of patient safety culture is widely known and used in Swedish healthcare in different contexts [18, 19], and was therefore chosen by the improvement group. The introduction of the Green Line reflections was less likely to improve patient safety culture. However, the hope for that was one of the reasons behind the project for the management of the ward, and the improvement group. Therefore it was found most true and honest to the project and the workplace to include this in the study.

When the interviews were conducted, there had been no safety huddles for a while, which may have affected the answers. It is difficult to draw conclusions from comparisons (surveys) over time when the conditions are constantly changing; there were changes in the local context and in the design of the improvement work, which may have affected the results. The response rate was quite low in the survey responses; hence, conclusions have to be drawn carefully. However, the survey was supplemented with interviews and the results were largely consistent across both methods.

The first author's pre-understanding from being a quality and patient safety developer at the department of paediatrics and from taking an active part in the improvement work may have affected the results. Pre-understanding can also be important in the analysis and interpretation of data and can contribute to in-depth knowledge and understanding [36].

## Conclusions

Based on this study´s results, it may be difficult to introduce reflections based on learning from everything that happens, including when things go well (Safety-II) into patient safety huddles. Careful planning is important for such interventions to be able to succeed. To make the reflections better, it is important to have support from managers, and for those who lead the safety huddles to have knowledge of the theories underpinning the Safety-II approach. For the participants, there needs to be an open and permissive climate, a plan to ensure that all professions can participate, and stable conditions in management and support of the safety huddles for them to be experienced as valuable for learning. Further studies are needed to understand how Safety-II-inspired safety huddles are best implemented and to determine whether increased understanding amongst employees of the purpose of the huddles may contribute to better patient safety and an improved patient safety culture.

## Supplementary Information

**Additional file 1.** PDSA Green line 2018. The first PDSA-circle described in the improvement work.

**Additional file 2.** Questionnaire. Contains the cross-questions and open-ended question used in the questionnaires in the study.

**Additional file 3.** Invitation to participate in study in web survey. Invitation to participate with information regarding the questionnaire web survey.

**Additional file 4.** Invitation to participate in interview. Invitation to participate with information regarding the interviews.

**Additional file 5.** Question guide. Question guide for interviews.

**Additional file 6.** Summary the Green line 2019 example. Example of the every month compilation, number of participants, profession, colour classification and what potentials the conversations could be classified within.

**Additional file 7.** Example of practical improvements. Example of improvements as a result from the reflections.

**Additional file 8.** Themes codes and meaning units. Themes and codes with examples of meaning units from the analysis of the interviews.

## Data Availability

The datasets generated and analysed during the current study are not publicly available due to the ethical approval but are available from the corresponding author on reasonable request.
