# Supplementary material for: Experience of learning from everyday work in daily safety huddles—a multi-method study
Source: BMC Health Serv Res. 2022 Aug 30;22:1101. doi: 10.1186/s12913-022-08462-9 (PMC9424837; doi:10.1186/s12913-022-08462-9)
Supplement: Supplementary file 1 — Additional file 1. PDSA Green line 2018. The first PDSA-circle described in the improvement work. [file 12913_2022_8462_MOESM1_ESM.pdf]

PGSA

Activities, process: The neonatal care unit

Area for improvement: Reflections for learning

Change to test: Introduce the Green line reflection

Responsible: HR, HEF, LE, AL, SL, KW

Additional file 1.

Start date: Autumn 2018

Act 4:

- Possible revision after pilot test and planning for the continuation
- Act based on what comes up in the reflection

Plan 1:

- Plan for testing and introduction of the Green Line reflections on neo, with a focus on learning based on the daily work when it goes well
- Introduction group is formed
- Plan for time and implementation

Study 3:

- Evaluate after pilot tests in weeks 41 and 42
- Follow the process and topics for reflection and learning
- Questionnaire responses are followed. Survey before introduction and approximately every three months during the first year to follow developments at the unit
- In-depth reflection once a month – documented
- Master's thesis? Capturing the significance of reflection for nursing, how is it affected?

Do 2:

- Spread information to the unit's staff via newsletters sent in week 40. LE and AL are responsible.
- A questionnaire is prepared and sent out before the start and approximately every three months
- Prepare material, Green Line template, template to document in, instructions for those who hold the reflection moment
- Prepare the bulletin board and binder to gather information
- Plan for pilot tests in weeks 41 and 42
