# Supplementary material for: Experience of learning from everyday work in daily safety huddles—a multi-method study
Source: BMC Health Serv Res. 2022 Aug 30;22:1101. doi: 10.1186/s12913-022-08462-9 (PMC9424837; doi:10.1186/s12913-022-08462-9)
Supplement: Supplementary file 2 — Additional file 2. Questionnaire. Contains the cross-questions and open-ended question used in the questionnaires in the study. [file 12913_2022_8462_MOESM2_ESM.pdf]

Region Jönköpings län

Additional file 2.

We would like you to respond to the following statements. These are issues that can reflect a sustainable safety commitment. We use this survey to follow the introduction of "Green Line Reflections". The questionnaire contains 14 cross-questions and one open-ended question where you can write your own comments if you want. There are also two questions about the Green Line reflection.

Your answer is handled anonymously.

Regards

1. I who answer is

- ☐ Doctor
- ☐ Nurse
- ☐ Assistant nurse
- ☐ Other occupational group

2. How long have you worked at the department of paediatrics?
year

- ☐ ≤2 year
- ☐ >2-≤5 year
- ☐ >5-≤10 year
- ☐ >10 year

3. My boss provides conditions for conducting safe care. (Safe care means a low incidence of patient injuries that can be avoided)

- ☐ 1. I totally disagree
- ☐ 2. I disagree
- ☐ 3. I neither agree or disagree
- ☐ 4. I agree
- ☐ 5. I fully agree

4. At my workplace, we learn from what works well.

- ☐ 1. I totally disagree
- ☐ 2. I disagree
- ☐ 3. I neither agree or disagree
- ☐ 4. I agree
- ☐ 5. I fully agree

5. At my workplace, we always act on the risks we see

- ☐ 1. I totally disagree
- ☐ 2. I disagree
- ☐ 3. I neither agree or disagree
- ☐ 4. I agree
- ☐ 5. I fully agree

6. At my workplace, improvements are always made after negative events. (Negative event is an event that causes something unwanted).

- ☐ 1. I totally disagree
- ☐ 2. I disagree
- ☐ 3. I neither agree or disagree
- ☐ 4. I agree
- ☐ 5. I fully agree

7. I point out when I think something is about to go wrong.

- ☐ 1. I totally disagree
- ☐ 2. I disagree
- ☐ 3. I neither agree or disagree
- ☐ 4. I agree
- ☐ 5. I fully agree

8. I dare to talk about my mistakes.

- ☐ 1. I totally disagree
- ☐ 2. I disagree
- ☐ 3. I neither agree or disagree
- ☐ 4. I agree
- ☐ 5. I fully agree

9. I am always well received at my workplace when I need help.

- ☐ 1. I totally disagree
- ☐ 2. I disagree
- ☐ 3. I neither agree or disagree
- ☐ 4. I agree
- ☐ 5. I fully agree

10. At my workplace, we have a well-functioning collaboration with other units in the department of paediatrics and other departments at the hospital.

- ☐ 1. I totally disagree
- ☐ 2. I disagree
- ☐ 3. I neither agree or disagree
- ☐ 4. I agree
- ☐ 5. I fully agree

11. At my workplace, we adapt the work so that safety is maintained when conditions change.

- ☐ 1. I totally disagree
- ☐ 2. I disagree
- ☐ 3. I neither agree or disagree
- ☐ 4. I agree
- ☐ 5. I fully agree

12. At my workplace, we have routines and working methods that allow us to be prepared for challenges that may arise.
(Question 12 in the questionnaire was not included in the study, see Methods in the article).

- ☐ 1. I totally disagree
- ☐ 2. I disagree
- ☐ 3. I neither agree or disagree
- ☐ 4. I agree
- ☐ 5. I fully agree

13. I would feel safe if a close relative was cared for at my workplace.

- ☐ 1. I totally disagree
- ☐ 2. I disagree
- ☐ 3. I neither agree or disagree
- ☐ 4. I agree
- ☐ 5. I fully agree

14. At my workplace, we offer parents / relatives the opportunity to be involved in our patient safety work.

- ☐ 1. I totally disagree
- ☐ 2. I disagree
- ☐ 3. I neither agree or disagree
- ☐ 4. I agree
- ☐ 5. I fully agree

15. The green line reflections lead to a learning

- ☐ 1. I totally disagree
- ☐ 2. I disagree
- ☐ 3. I neither agree or disagree
- ☐ 4. I agree
- ☐ 5. I fully agree

16. Comments on the Green Line reflections? Enter positive as well as negative.
