# Supplementary material for: Experience of learning from everyday work in daily safety huddles—a multi-method study
Source: BMC Health Serv Res. 2022 Aug 30;22:1101. doi: 10.1186/s12913-022-08462-9 (PMC9424837; doi:10.1186/s12913-022-08462-9)
Supplement: Supplementary file 3 — Additional file 3. Invitation to participate in study in web survey. Invitation to participate with information regarding the questionnaire web survey. [file 12913_2022_8462_MOESM3_ESM.pdf]

Region Jönköpings län

Additional file 3

Information about the web survey:

Information to study participants

We want to ask you if you want to participate in a research project. In this document you get information about the project and what it means to participate.

What kind of project are you and why do you want me to participate?

The current scientific study is conducted to describe the experiences of the introduction of the Green Line Reflections and how this method of reflection may have affected the patient safety culture in a workplace. All employees working at the neonatal care unit, Ryhov County Hospital, Jönköping in the autumn of 2020 are invited to participate in the study.

How is the study done?

The study consists of a web survey with 17 questions that are conducted during working hours and takes about 5 minutes to answer. The questionnaire is sent out via work e-mail in the esMaker system, the same system that Region Jönköping County uses when conducting employee surveys. The study will be carried out in the autumn of 2020. If you have not participated in any reflection session with the Green Line, you can answer the questions based on how you think about patient safety issues.

What happens to my information?

The project will collect and register the information provided in the survey. Data on participating activities and researchers are collected in accordance with the General Data Protection Regulation (GDPR). The person responsible for personal data is Johan Cederlund, RJL (010 - 242 45 30, e-mail: johan.cederlund@rjl.se). Your answers will be processed so that unauthorized persons cannot take part in them. The results will be compiled so that no individual can be identified. The study material will be saved for ten years on password-protected servers within the Jönköping County Region.

Participation is voluntary

Your participation is voluntary and you can choose to cancel your participation at any time before submitting the questionnaire. The Es-Maker system is structured so that your answers cannot be linked to your work email, which means that we cannot subsequently delete your submitted answers to the survey questions. If you choose not to participate or want to cancel your participation, you do not have to state why, nor will it affect your duties or your

How do I get information about the results of the study?

The results will also be presented at department level as well as at regional, national and international conferences / meetings. The results will also be published as an article in an international scientific journal.

If you want further information or have questions or concerns about the study, you can contact those responsible for the study.

Responsible for the study

Axel Ros	Karina Wahl
Chief medical officer Region Jönköping County	Quality, patient safety developer Department of Paediatrics
axel.ros@rjl.se	karina.wahl@rjl.se
010-242 13 47	010-242 12 23
