# Supplementary material for: Experience of learning from everyday work in daily safety huddles—a multi-method study
Source: BMC Health Serv Res. 2022 Aug 30;22:1101. doi: 10.1186/s12913-022-08462-9 (PMC9424837; doi:10.1186/s12913-022-08462-9)
Supplement: Supplementary file 4 — Additional file 4. Invitation to participate in interview. Invitation to participate with information regarding the interviews. [file 12913_2022_8462_MOESM4_ESM.pdf]

Region Jönköpings län

Additional file 4

Invitation to participate in study:

Information about the study

We want to ask you if you want to participate in a research project. In this document you get information about the project and what it means to participate.

What kind of project is this and why we ask you to participate

The current scientific study is conducted to describe the experiences of the introduction of the Green Line Reflections and how this method of reflection may have affected the patient safety culture in a workplace. The study will be carried out in the autumn 2020 as an interview study with employees at the neonatal care unit, Länssjukhuset Ryhov, Jönköping. Those who participate in the interviews belong to different professional groups and have different experiences of working in paediatric care. Everyone interviewed has participated in at least one reflection session with the Green Line.

How is the study done?

If you choose to participate, you will be invited to an interview with Karina Wahl, quality and patient safety developer, as interviewer. The interview is recorded on tape and takes about 30 minutes. Each interview is provided with a code and your name will not be mentioned during the recording. At the beginning of the interview, the interviewer asks questions about your profession, how long you have worked at the Department of Paediatrics and how often you have participated in the Green Line Reflections. This information is written down on a separate form and with the same code as the interview but without name or other personal information

What happens to my information?

The project will collect and record the information provided at the interview. Data on participating activities and researchers are collected in accordance with the General Data Protection Regulation (GDPR). The person responsible for personal data is Johan Cederlund, RJL (010 - 242 45 30, e-mail: johan.cederlund@rjl.se). Your answers will be processed so that unauthorized persons cannot take part in them. The study material will be stored for ten years on password-protected servers in the Jönköping County Region and in locked cabinets at the Department of Paediatrics and treated so that no unauthorized persons can access the material. The interview material will be analyzed with qualitative methodology and presented at a general level and with occasional illustrative quotes. When presenting the results, it will not be clear which people have participated in individual interviews or what individuals have said.

Participation is voluntary

Your participation is voluntary and you can choose to cancel the participation at any time until the interview ends. If you choose not to participate or want to cancel your participation, you do not need to state why. For research ethics reasons, all material collected must be saved if the study is reviewed afterwards. As the material is saved without connection to who said what, we can not subsequently delete the reflections you have shared with you.

How do I get information about the results of the study?

The results will also be presented at department level as well as at regional, national and international conferences / meetings. The results will also be published as an article in an international scientific journal.

If you want further information or have questions or concerns about the study, you can contact those responsible for the study.

Responsible for the study:

Axel Ros
Chief medical officer Region Jönköping County
axel.ros@rjl.se
010-242 13 47

Karina Wahl
Quality, patient safety developer Department of Paediatrics
karina.wahl@rjl.se
010-242 12 23
