# Supplementary material for: Experience of learning from everyday work in daily safety huddles—a multi-method study
Source: BMC Health Serv Res. 2022 Aug 30;22:1101. doi: 10.1186/s12913-022-08462-9 (PMC9424837; doi:10.1186/s12913-022-08462-9)
Supplement: Supplementary file 5 — Additional file 5. Question guide. Question guide for interviews. [file 12913_2022_8462_MOESM5_ESM.pdf]

Additional file 5

Question guide

Demographic issues:

- How long have you worked at the Department of Paediatrics?
- In which profession?
- How often have you participated in any reflection sessions, approximately 1 time / week, approximately 1 time / month, less often than that?

Interview questions:

- When we talk about patient safety - what do you think about then?
- What situations / events do you think it is possible to learn something from?
- Can you tell us about something you or someone else brought up during a reflection session? Something that went well? Something that went wrong? What happened in reality and at the moment of reflection?
- Other views, experiences?
