# Supplementary material for: Experience of learning from everyday work in daily safety huddles—a multi-method study
Source: BMC Health Serv Res. 2022 Aug 30;22:1101. doi: 10.1186/s12913-022-08462-9 (PMC9424837; doi:10.1186/s12913-022-08462-9)
Supplement: Supplementary file 6 — Additional file 6. Summary the Green line 2019 example. Example of the every month compilation, number of participants, profession, colour classification and what potentials the conversations could be classified within. [file 12913_2022_8462_MOESM6_ESM.pdf]

Summary The Green Line - reflection for learning 2019 - example

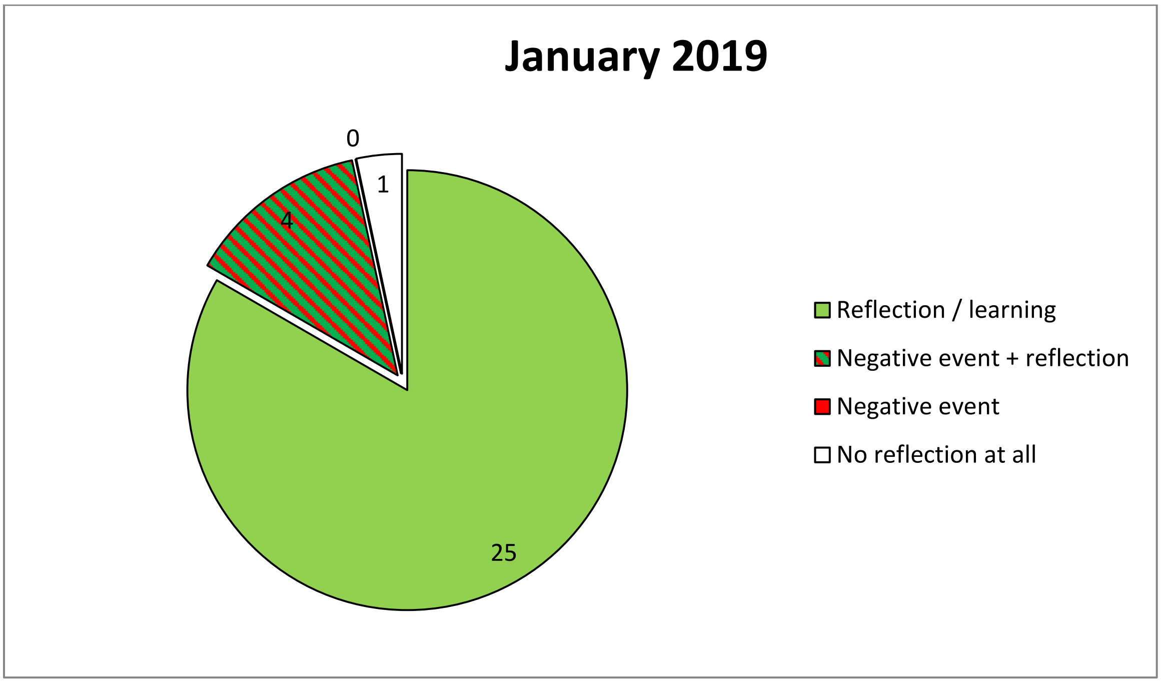

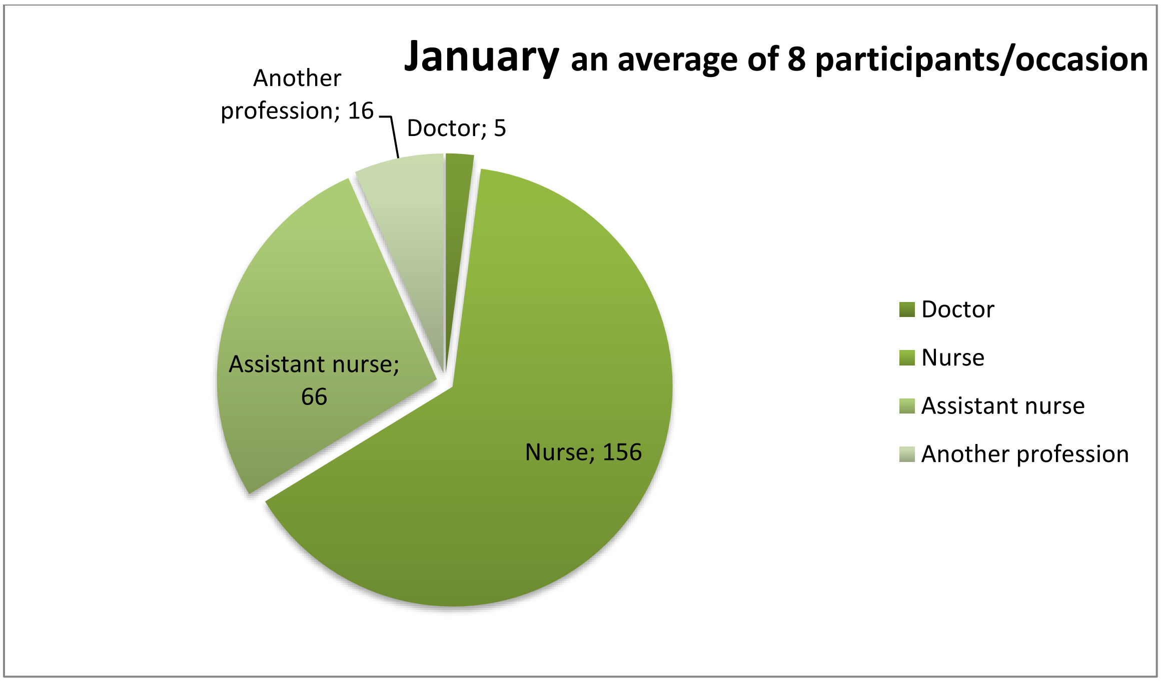

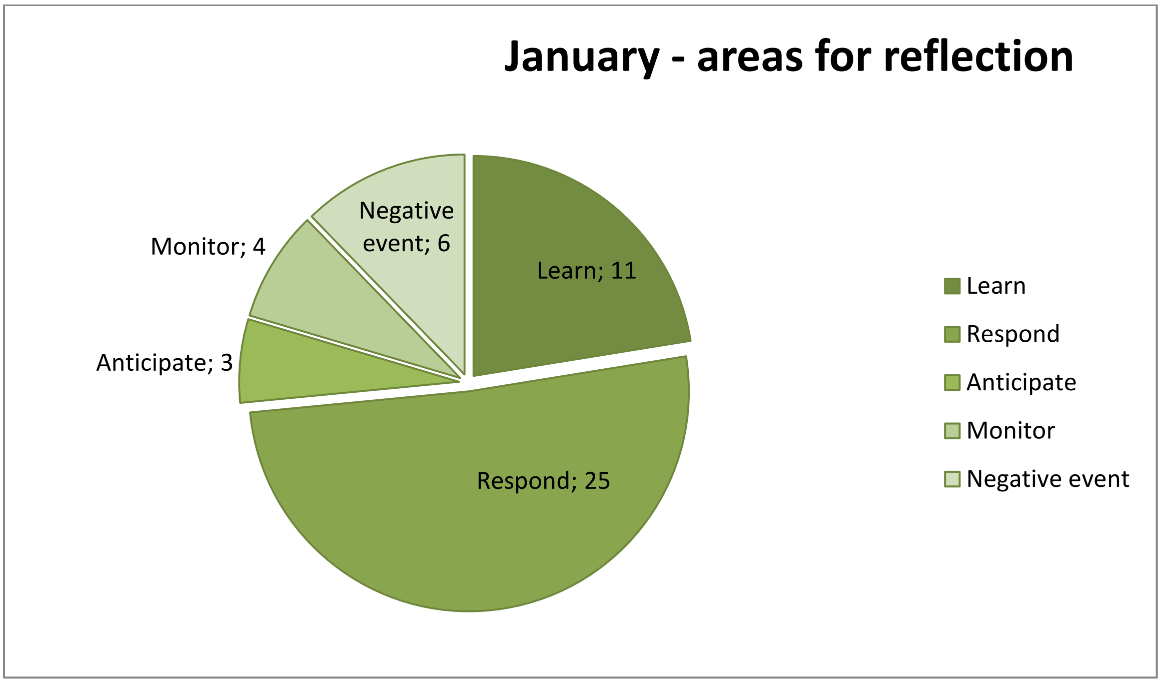
January - areas for reflection
Negative event; 6
Monitor; 4
Learn; 11
Anticipate; 3
Respond; 25
Learn
Respond
Anticipate
Monitor
Negative event

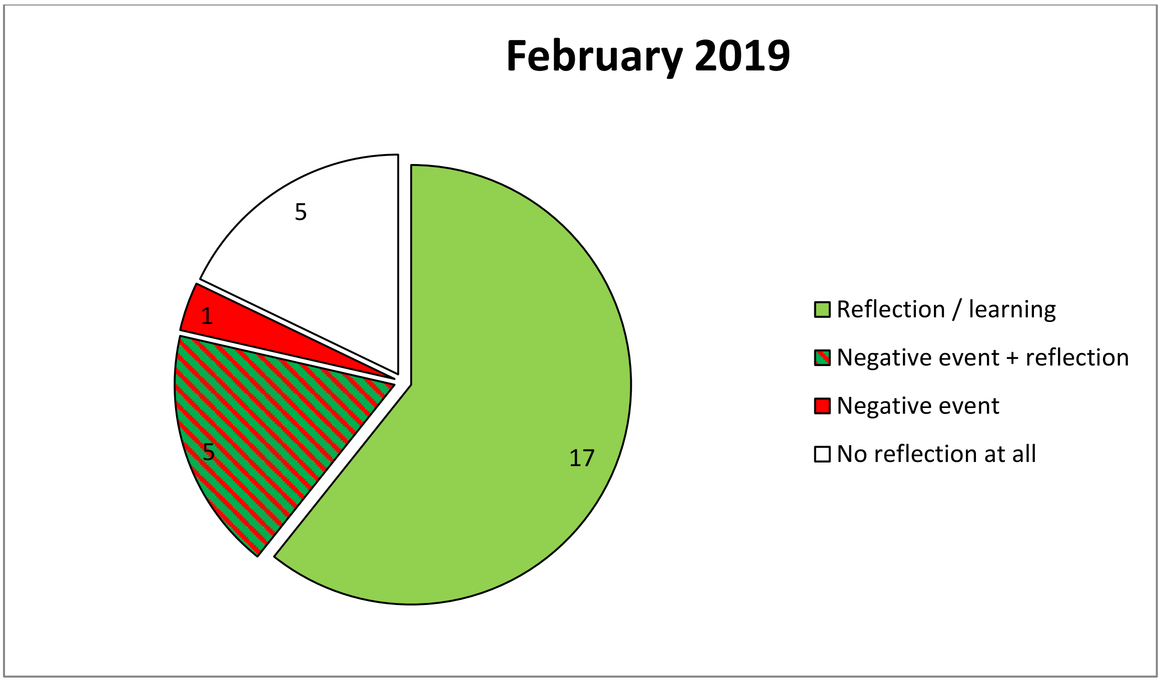
February 2019
5
1
5
17
Reflection / learning
Negative event + reflection
Negative event
No reflection at all

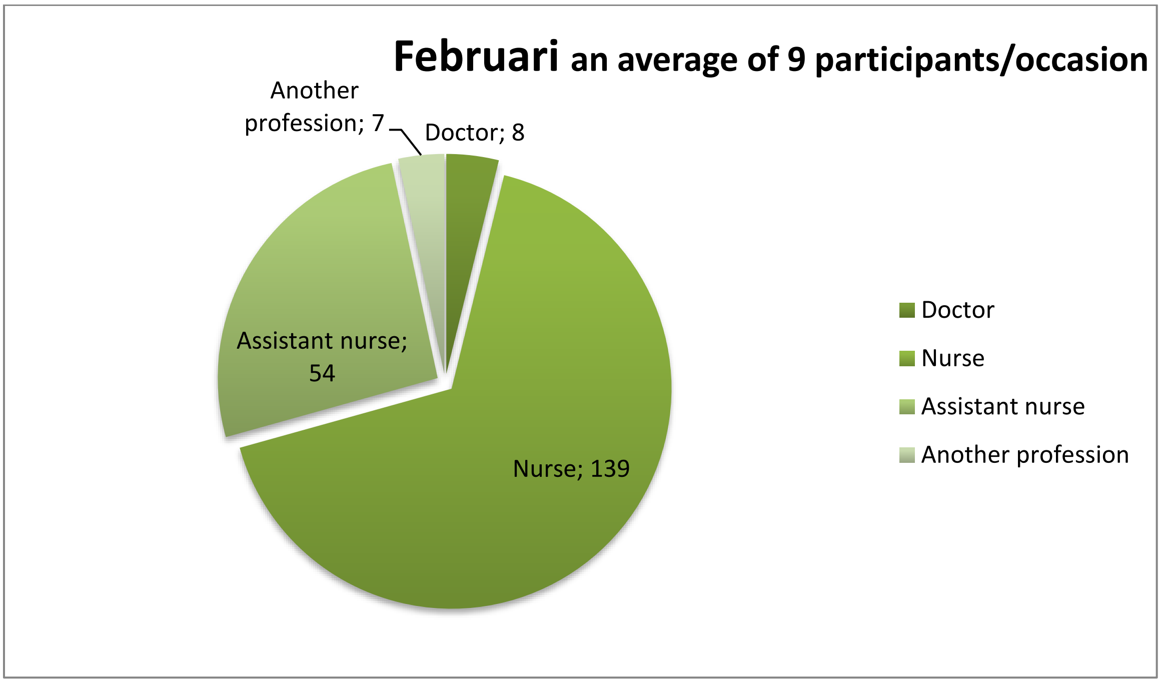
Februari an average of 9 participants/occasion
Another profession; 7
Doctor; 8
Assistant nurse; 54
Nurse; 139
Doctor
Nurse
Assistant nurse
Another profession

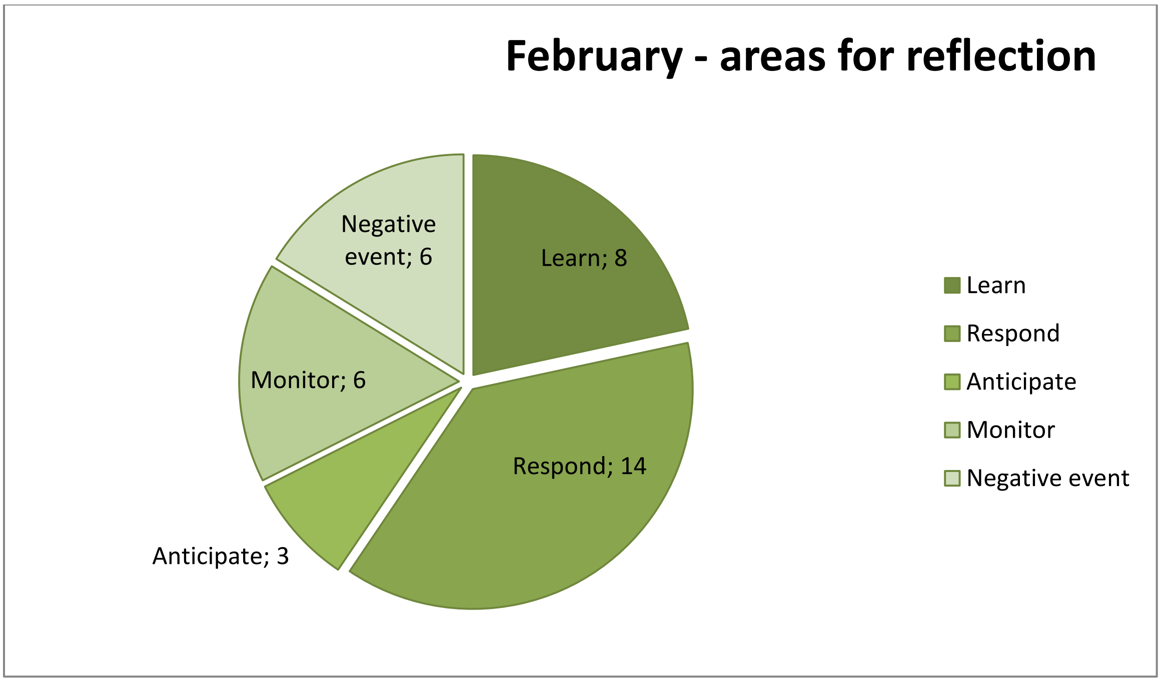
February - areas for reflection
Negative event; 6
Learn; 8
Monitor; 6
Respond; 14
Anticipate; 3
Learn
Respond
Anticipate
Monitor
Negative event

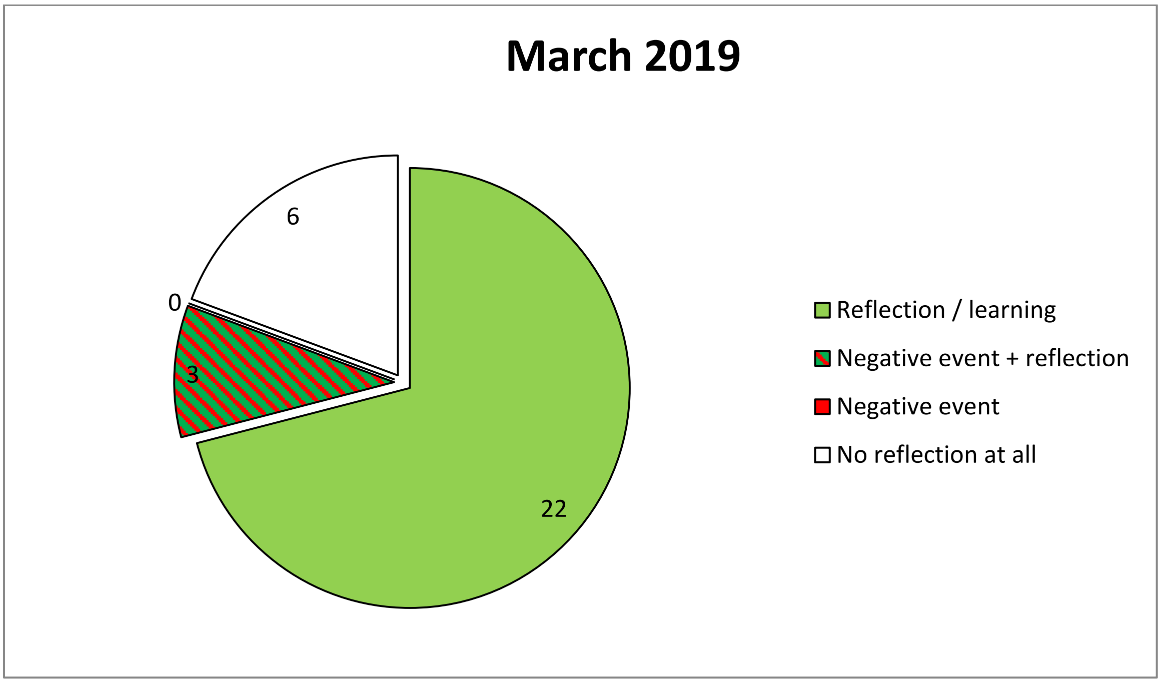
March 2019
6
0
3
22
Reflection / learning
Negative event + reflection
Negative event
No reflection at all

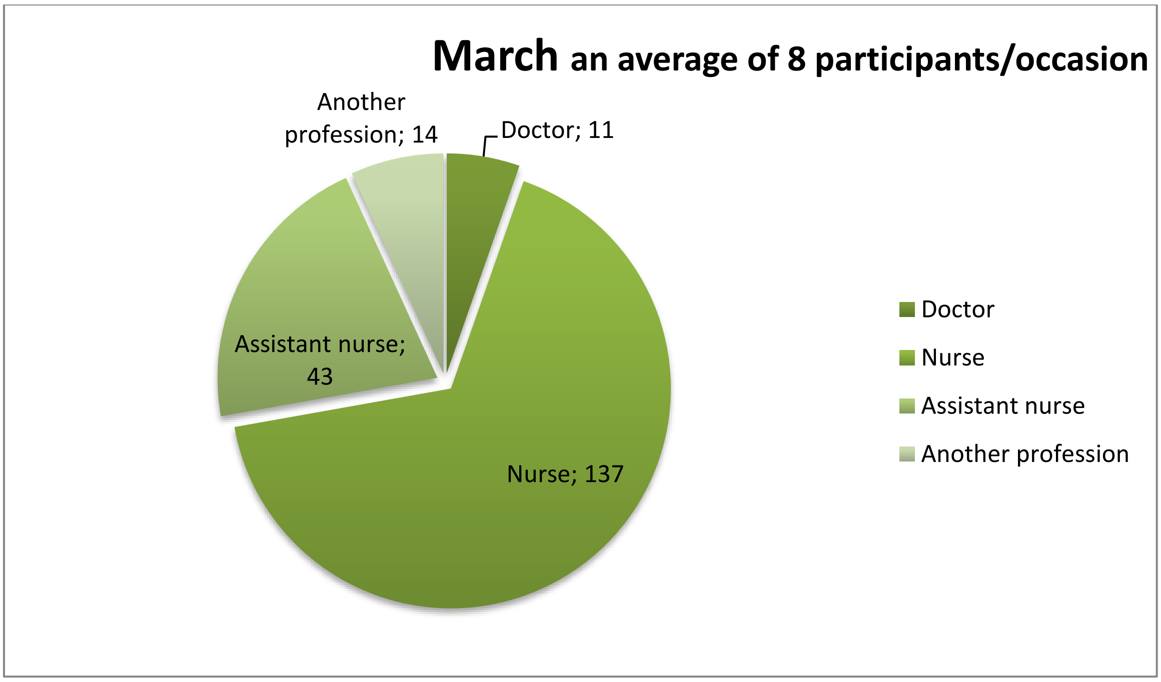
March an average of 8 participants/occasion
Another profession; 14
Doctor; 11
Assistant nurse; 43
Nurse; 137
Doctor
Nurse
Assistant nurse
Another profession

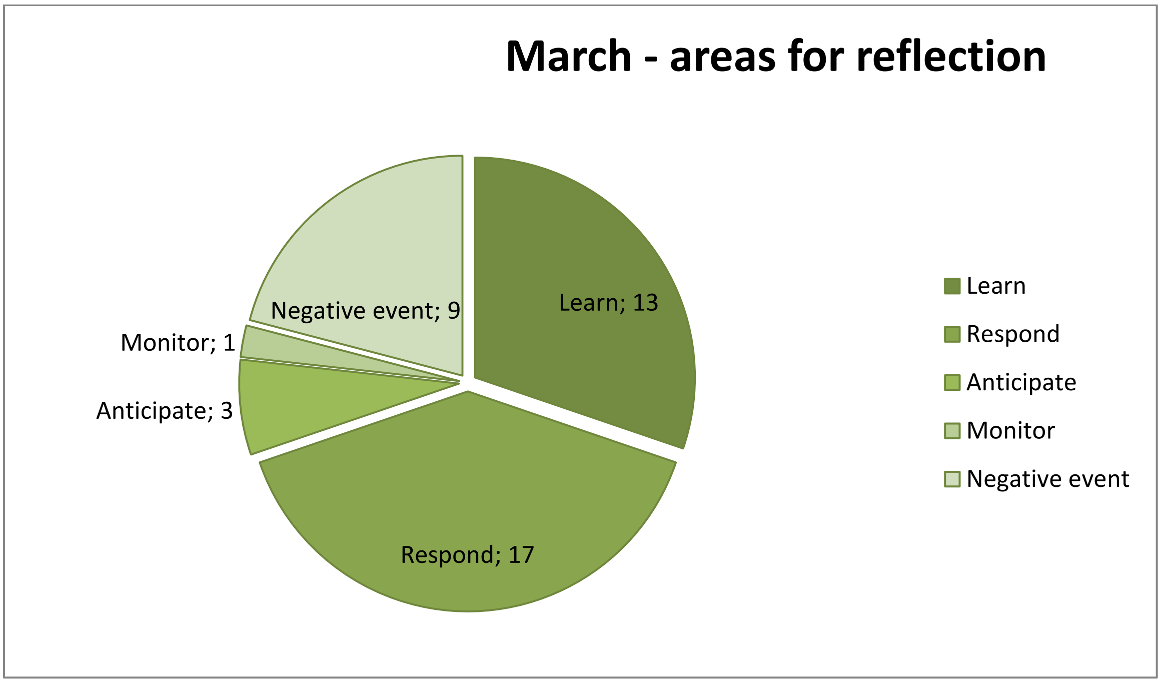
March - areas for reflection
Negative event; 9
Learn; 13
Monitor; 1
Anticipate; 3
Respond; 17
Learn
Respond
Anticipate
Monitor
Negative event

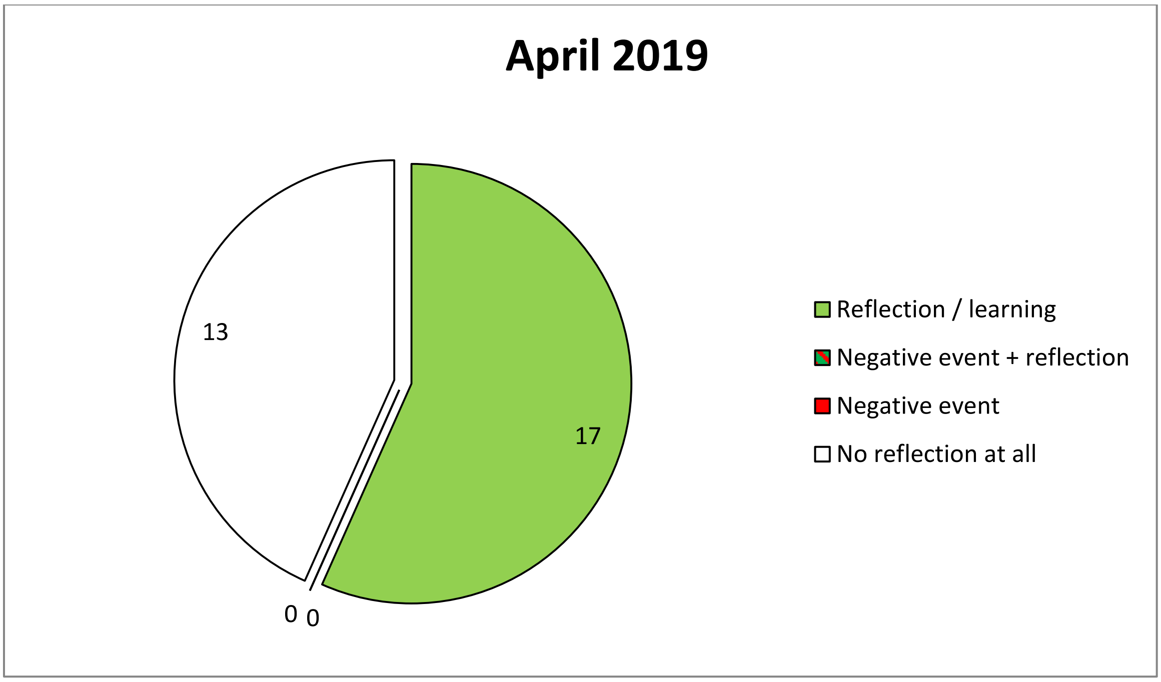
April 2019
13
17
0
0
Reflection / learning
Negative event + reflection
Negative event
No reflection at all

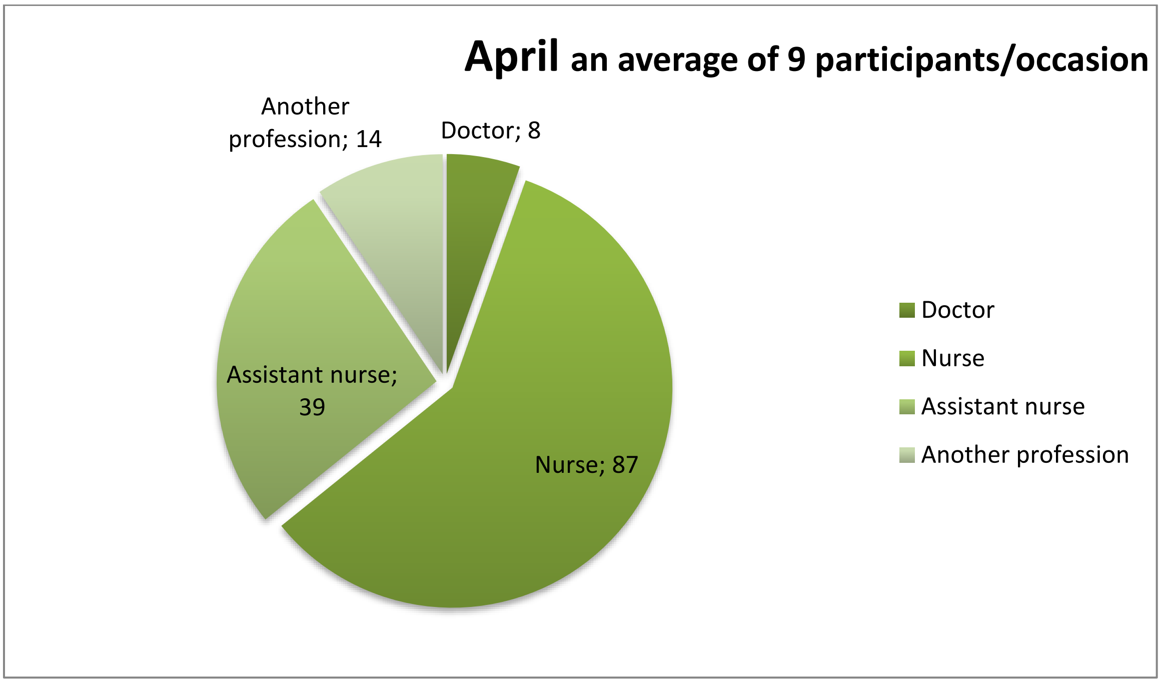
April an average of 9 participants/occasion
Another profession; 14
Doctor; 8
Assistant nurse; 39
Nurse; 87
Doctor
Nurse
Assistant nurse
Another profession

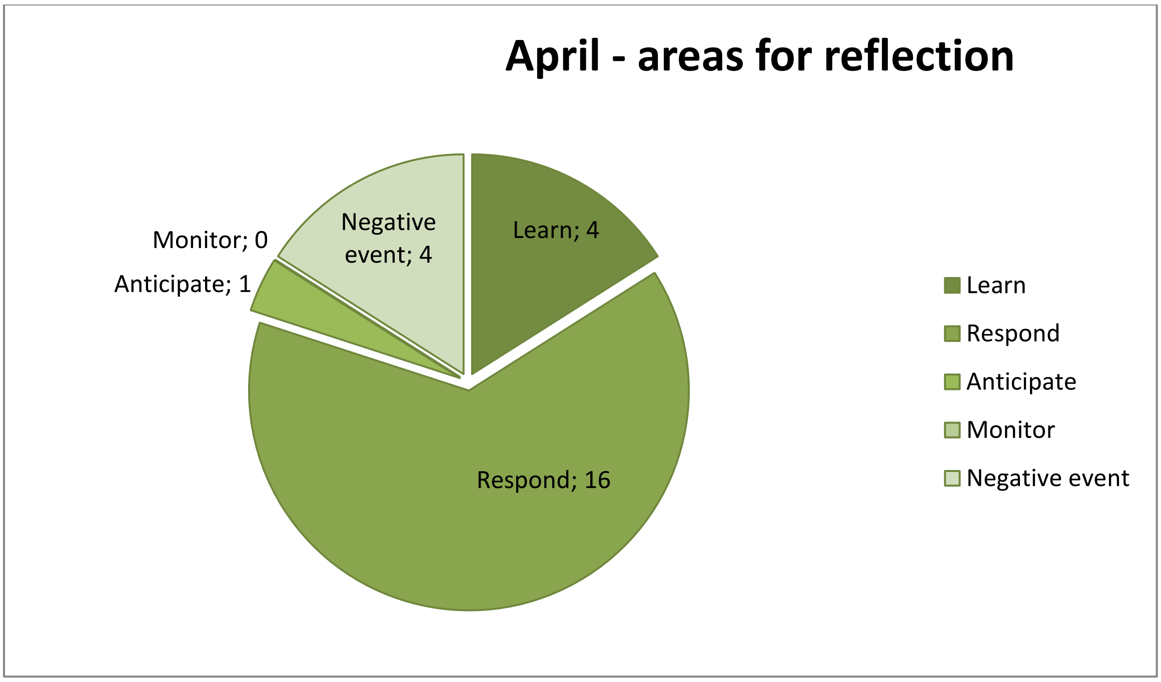
April - areas for reflection
Monitor; 0
Anticipate; 1
Negative event; 4
Learn; 4
Respond; 16
Learn
Respond
Anticipate
Monitor
Negative event

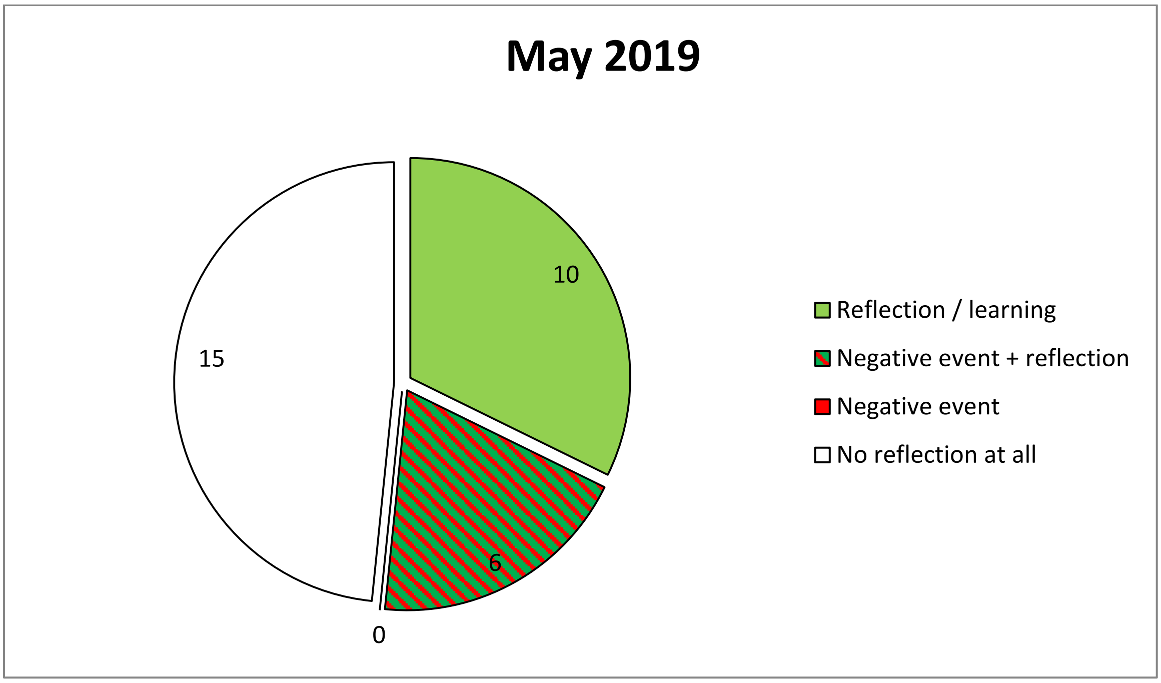
May 2019
10
15
6
0
Reflection / learning
Negative event + reflection
Negative event
No reflection at all

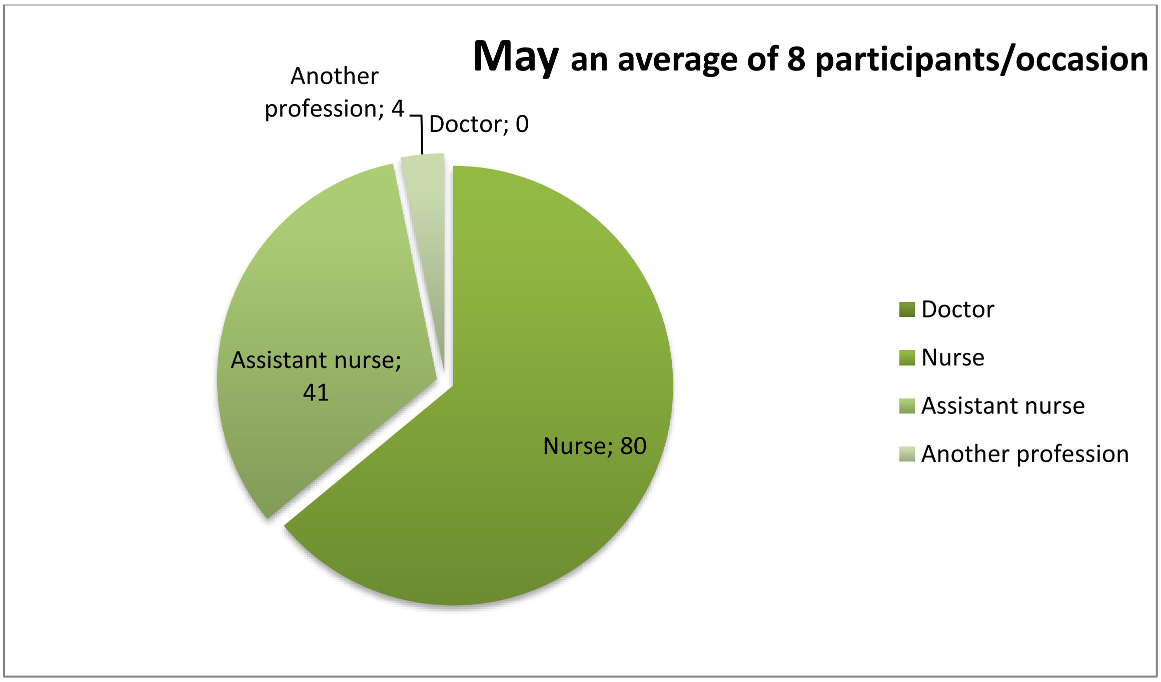
May an average of 8 participants/occasion
Another profession; 4
Doctor; 0
Assistant nurse; 41
Nurse; 80
Doctor
Nurse
Assistant nurse
Another profession

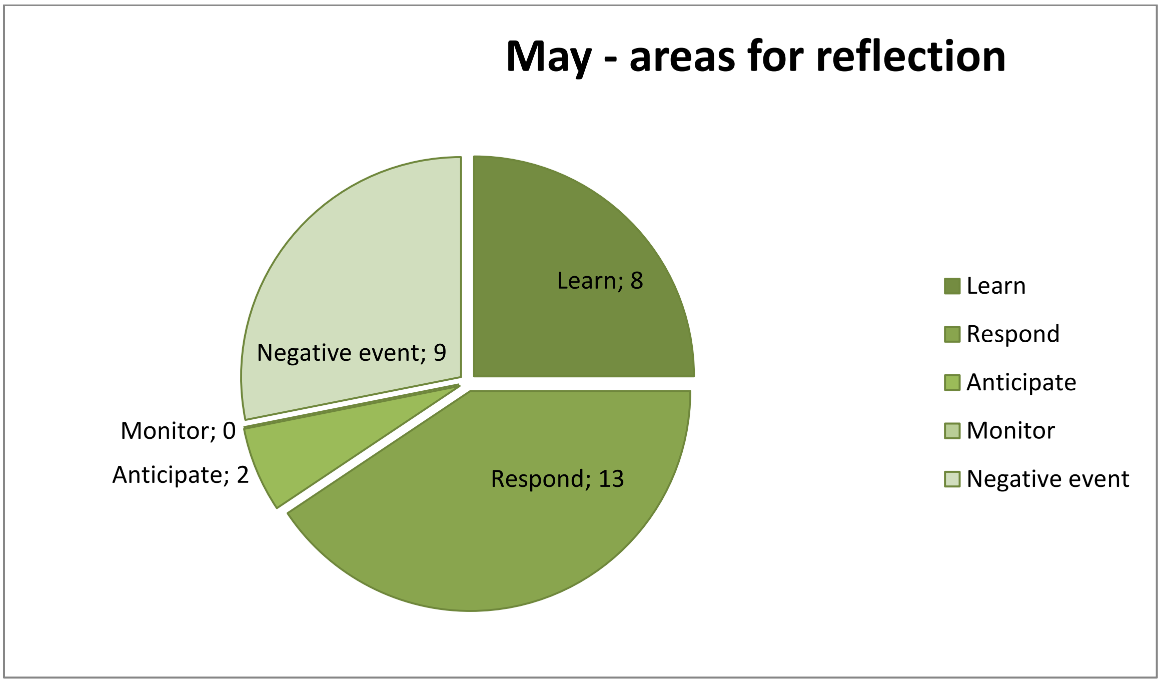
May - areas for reflection
Learn; 8
Negative event; 9
Monitor; 0
Anticipate; 2
Respond; 13
Learn
Respond
Anticipate
Monitor
Negative event
