# Supplementary material for: Experience of learning from everyday work in daily safety huddles—a multi-method study
Source: BMC Health Serv Res. 2022 Aug 30;22:1101. doi: 10.1186/s12913-022-08462-9 (PMC9424837; doi:10.1186/s12913-022-08462-9)
Supplement: Supplementary file 7 — Additional file 7. Example of practical improvements. Example of improvements as a result from the reflections. [file 12913_2022_8462_MOESM7_ESM.pdf]

Some example of practical improvements as a result from the reflections;

They started with "Tuesday reviews" practical exercise of equipment, such as pleural pump and routines including food routines such as handling of breast milk, dates of drugs to avoid throwing - reminder.

Distribute duties better, e.g. responsibility for the phone in the unit.

The wording in the SMS, in the event of a shortage of people, was changed so that staff understood what was going on.
