# Supplementary material for: Experience of learning from everyday work in daily safety huddles—a multi-method study
Source: BMC Health Serv Res. 2022 Aug 30;22:1101. doi: 10.1186/s12913-022-08462-9 (PMC9424837; doi:10.1186/s12913-022-08462-9)
Supplement: Supplementary file 8 — Additional file 8. Themes codes and meaning units. Themes and codes with examples of meaning units from the analysis of the interviews. [file 12913_2022_8462_MOESM8_ESM.pdf]

Additional file 8

Themes and codes with examples of meaning units from the analysis of the interviews.

**Theme**	**Codes**	**Meaning units**
Supporting factors	Seeing benefits with reflection	*“I really think it's great …. it's not only mistakes that should be noticed, you can learn a lot from each other, everyone has different experiences….”*
		"It's such a scattered department you may not even see others throughout the shift... I think there will be a little more cohesion in the group with reflections ..."
	Learning from what happens	*“it is difficult because…for everything in life really, if you do not hear anything, then it is probably often good… you are only told the bad things”*
		“No one dared to say anything that was negative”
	Finding improve-ments for a rewarding reflection	*"… Someone who is clear about the purpose and who agrees with the purpose, I think so, not just someone who is set to lead that reflection"*
		“it became more lively when we started using focus topics”.
		“…they became more inspired... the (reflection) leader must have the ability to angle the questions”.
		“…that the managers try to participate and are interested and also think it is important”.
Hindering factors	Seeing difficulties with reflection	*"..There was often a lot of repetition, it was the same thing. And everything that becomes the same thing becomes very boring”,*
		“Feedback is given but stays there ……..it may need to reach other people … or make improvements… often it stays in the small group… and the challenge may continue to bother you”
	The impact of the work climate	*“we should have a slightly more open climate in our department …. the attitude of some in the staff group may be …. judges a little too easily sometimes”*
		“ …when you are new, you are invisible”
		“Managers must be involved in the Green Line and support an open climate...”
